# Increased Response to β_2_-Adrenoreceptor Stimulation Augments Inhibition of I_Kr_ in Heart Failure Ventricular Myocytes

**DOI:** 10.1371/journal.pone.0046186

**Published:** 2012-09-28

**Authors:** Hegui Wang, Yanhong Chen, Hongjun Zhu, Sen Wang, Xiwen Zhang, Dongjie Xu, Kejiang Cao, Jiangang Zou

**Affiliations:** 1 Department of Cardiology, Yijishan Hospital of Wannan Medical College, Wuhu, China; 2 Department of Cardiology, the First Affiliated Hospital, Nanjing Medical University, Nanjing, China; University of Torino, Italy

## Abstract

**Background:**

Increasing evidence indicates that the rapid component of delayed rectifier potassium current (I_Kr_) is modulated by α- and β-adrenergic stimulation. However, the role and mechanism regulating I_Kr_ through β_2_-adrenoreceptor (β-AR) stimulation in heart failure (HF) are unclear.

**Methodology/Principal Findings:**

In the present study, we investigated the effects of fenoterol, a highly selective β_2_-AR agonist, on I_Kr_ in left ventricular myocytes obtained from control and guinea pigs with HF induced by descending aortic banding. I_Kr_ was measured by using whole cell patch clamp technique. In control myocytes, superfusion of fenoterol (10 µM) caused a 17% decrease in I_Kr_. In HF myocytes, the same concentration of fenoterol produced a significantly greater decrease (33%) in I_Kr_. These effects were not modified by the incubation of myocytes with CGP-20712A, a β_1_-AR antagonist, but were abolished by pretreatment of myocytes with ICI-118551, a β_2_-AR antagonist. An inhibitory cAMP analog, Rp-cAMPS and PKA inhibitor significantly attenuated fenoterol-induced inhibition of I_Kr_ in HF myocytes. Moreover, fenoterol markedly prolonged action potential durations at 90% (APD_90_) repolarization in HF ventricular myocytes.

**Conclusions:**

The results indicate that inhibition of I_Kr_ induced by β_2_-AR stimulation is increased in HF. The inhibitory effect is likely to be mediated through a cAMP/PKA pathway in HF ventricular myocytes.

## Introduction

Heart failure (HF) is associated with significant mortality, with nearly 50% of deaths occurring suddenly, primarily from ventricular tachycardia to ventricular fibrillation [1]. Sympathetic nerve activity is increased in HF [2]. It is widely accepted that the cardiac response to catecholamines is mediated primarily by β-adrenoreceptors (β-ARs). Arrhythmogenesis in HF is enhanced by β-adrenergic stimulation [3].

The human ether-a-go-go-related gene (hERG or *KCNH2*) [4] encodes the α subunit of the channel underlying I_Kr_ [5], which is crucial for the repolarization of cardiac action potentials (AP). I_Kr_ is modulated by catecholamines. There is increasing evidence that hERG/I_Kr_ channels are modulated by various G protein-coupled receptors including α- and β-ARs, which act through the intracellular signaling modulators cAMP, protein kinase A (PKA), and protein kinase C (PKC) [6], [7], [8], [9]. Stimulation of β-AR by 10 µM isoprenaline decreased I_Kr_ tail currents in guinea pig ventricular myocytes [7]. Similarly, PKA-mediated phosphorylation of expressed hERG channels significantly decreased hERG currents [10]. However, recent studies have revealed that I_Kr_ tail currents are enhanced by 100 nM isoprenaline in canine ventricular myocytes [11]. It is well known that β_1_-AR is downregulated and β_2_-AR is relatively preserved in HF. In addition, experimental studies have demonstrated that canine ventricular response to β_2_-agonists is increased in tachypacing failure [12]. The responsiveness of cardiac *L*-type calcium current to β_2_-AR stimulation is increased in rats with HF induced by ligation of the coronary artery [13]. However, the role and mechanism of β_2_-AR activation in I_Kr_ in HF have not been previously assessed. Accordingly, the present study was designed to examine the role and possible mechanisms of β_2_-AR activation in I_Kr_ in left ventricular (LV) myocytes from HF guinea pigs using the whole cell patch clamp technique.

## Results

### Validity of the Guinea Pig Model

Following 12 weeks of thoracic aortic banding, the ejection fraction (EF) and fractional shortening (FS) of the heart of guinea pigs were significantly decreased; LV end-diastolic diameter, LV end-systolic diameter, the QT interval, and corrected QT interval were significantly increased while there were no changes in heart rate (Table 1). Cell capacitance of HF LV myocytes (n = 35 cells; 20 animals) and control LV myocytes (n = 35 cells; 16 animals) was 163±7 and 121±3 pF, respectively (P<0.001). Cell capacitance was increased in HF LV myocytes. All guinea pigs with HF developed ascites and pleural effusion. Thus, the HF model of guinea pigs induced by pressure overload is valid.

**Table 1:** Echocardiographic and electrocardiographic data of control and heart failure guinea pigs.

Group	CTL(n = 10)	HF(n = 10)
EF (%)	80.97±0.28	67.88±2.66**
FS (%)	44.47±0.27	34.01±1.95**
LVEDd (mm)	6.25±0.91	7.75±1.32*
LVESd (mm)	3.51±0.61	5.28±0.48**
HR(beats/min)	243±11	260±12
QT(ms)	142.44±5.73	167.98±9.37*
QT_C_(ms)	284.43±10.76	327.48±8.55*

Values are mean±SEM. Control (CTL), sham-operated guinea pigs; EF, ejection fraction; FS, fractional shortening; LVEDd, left ventricle end-diastolic diameter; LVESd, left ventricle end-systolic diameter; HR, heart rate; QT, interval; QTc, corrected QT interval; n, number of guinea pigs. *p<0.05, **p<0.01 vs. CTL.

### HF Reduces I_kr_ Tail Current Density

**Figure 1:**
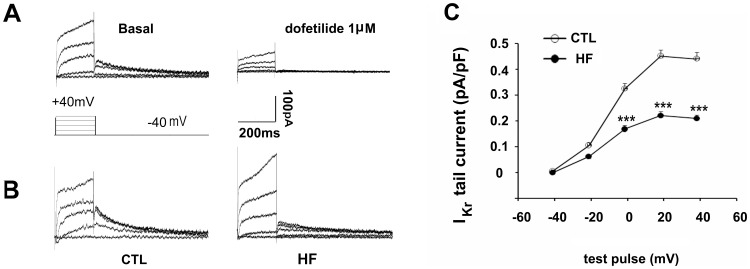
Changes of I_Kr_ in heart failure (HF). (A) Recording of I_kr_ tail current in a representative left ventricle (LV) myocyte before (left) and 10 min after exposure to dofetilide (1 µM) (right). (B) Representative tail traces of I_Kr_ in LV myocytes isolated from control (CTL, left) and HF guinea pigs (right). (C) The average current-voltage relationship of I_Kr_ plotted for control (n = 24 cells, 8 hearts) and HF (n = 33 cells, 10 hearts) myocytes (***p<0.001, HF vs. CTL). Test pulses were applied at various voltages from −40 to +40 mV (step width 20 mV, step duration 200 ms) before returning to −40 mV for tail current recording.

I_kr_ tail currents of guinea pig LV myocyte were completely abolished by the specific I_kr_ blocker, dofetilide (1 µm) (Figure 1A), indicating that I_kr_ tail currents were measured free of contamination by other currents under the given experimental conditions. The amplitude of I_Kr_ tail currents in HF myocytes was smaller than that of sham-operated controls (Figure 1B). Figure 1C shows the current–voltage (I–V) relationship of I_Kr_ tail current density. The I_Kr_ tail current density in HF myocytes was significantly lower than that in control myocytes, from 0 mV to positive potentials (at +40 mV, 0.21±0.01 pA/pF, n = 33, vs. 0.43±0.03 pA/pF, n = 24; p<0.001).

### I_kr_ Inhibition Induced by β_2_-AR Stimulation in HF Ventricular Myocytes

**Figure 2:**
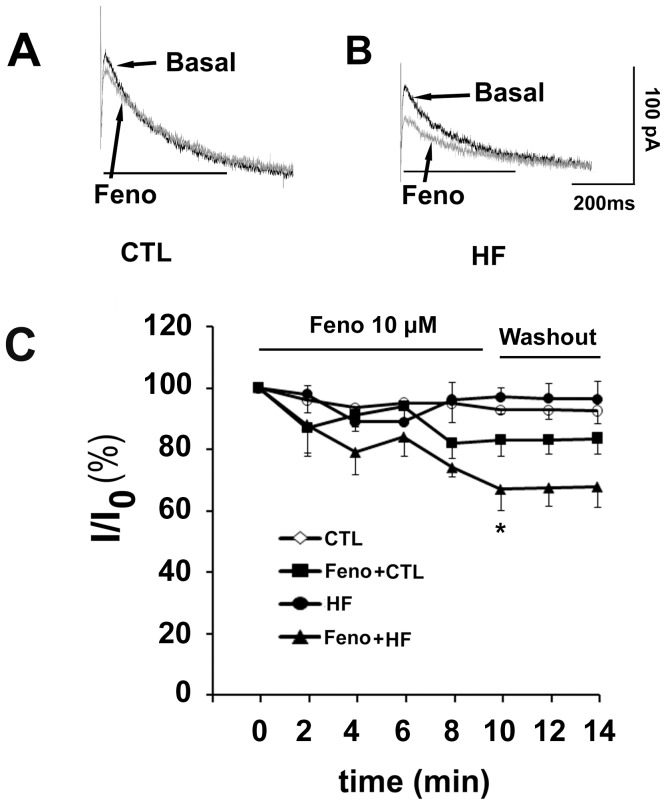
Effects of fenoterol (Feno) on I_kr_. (A and B) Superimposed tail current traces of I_kr_ recorded before and 10 min after application of 10 µM Feno in a control myocyte and a heart failure (HF) myocyte, respectively. (C) Time-dependence of current reduction by the β_2_-AR agonist fenoterol in control (n = 6 cells, 3 hearts) and HF myocytes (n = 6 cells, 4 hearts, *p<0.05, Feno+HF vs. HF). Current amplitudes were measured at the voltage of +40 mV.

In control myocytes, the selective β_2_-AR agonist fenoterol (10 µM) decreased I_kr_ tail currents by 17±5% (n = 6, vs. control myocytes in the absence of fenoterol, p = 0.11; Figure 2A and 2C). In contrast, fenoterol (10 µM) decreased I_kr_ tail currents by 33±7% in HF myocytes (n = 6, vs. HF myocytes in the absence of fenoterol, p<0.05; Figure 2B and 2C). The reduction of I_Kr_ tail current occurred within 2–4 min and reached saturation about 10 min after addition of fenoterol (10 µM) into the bath. After washout of fenoterol, I_kr_ tail current reached 83.5±4.5% and 67.8±6.7% of the control and HF value, respectively. The inhibitory effect of fenoterol was almost not reversible (Figure 2C).

### Effects of β_1_- and β_2_-AR Blockade on Fenoterol-induced Decrease in I_kr_

**Figure 3:**
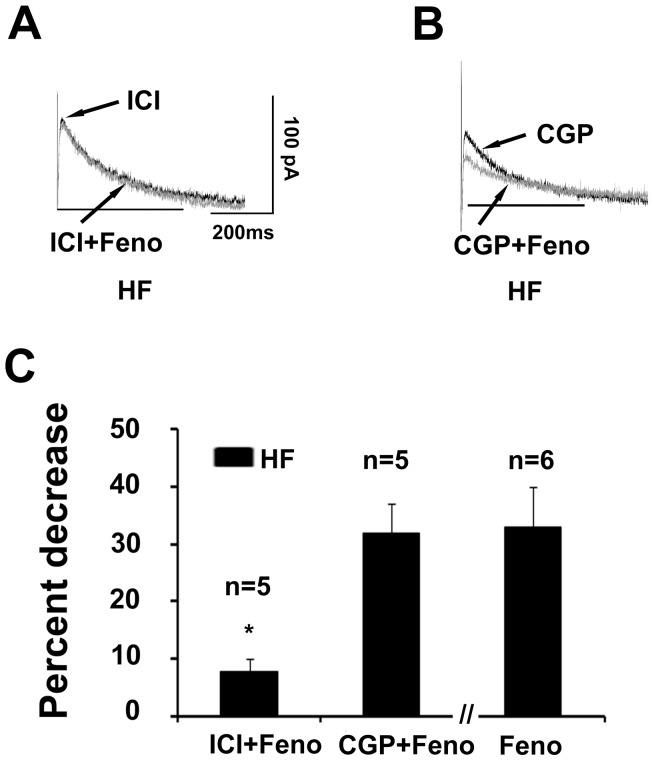
β_2_-AR mediates the inhibition of I_kr_ by fenoterol (Feno) in heart failure (HF) myocytes. (A and B) Superimposed tail current traces of I_kr_ recorded before and 10 min after application of 10 µM fenoterol in the presence of the selective β_2_-AR antagonist ICI118551 (ICI, 10 µM) and β_1_-AR antagonist CGP20712A (CGP, 10 µM) in a HF myocyte, respectively. (C) Summarized data for percent decrease in the amplitude of I_Kr_ tail current evoked by ICI plus fenoterol, CGP plus fenoterol, and fenoterol alone (n = 5 and 6 cells, 3 hearts, *P<0.05, Feno+ICI versus Feno). Current amplitudes were measured at the voltage of +40 mV.

We used the selective β_2_-AR antagonist ICI118551 to examine whether the I_Kr_ response to fenoterol was mediated through β_2_-AR in HF myocytes. When 10 µM ICI118551 was applied along with fenoterol, it almost totally prevented fenoterol-induced inhibition of I_Kr_ (fenoterol alone, 33±7% decrease, n = 6; fenoterol plus ICI118551, 8±3% decrease, n = 5; p<0.05; Figure 3A and 3C). However, in five HF myocytes, fenoterol decreased I_Kr_ by 32±5% in the presence of CGP20712A, a selective β_1_-AR antagonist (Figure 3B and 3C). Under these conditions, the present results supported the role of the β_2_-AR subtype in the modulation of I_Kr_ in HF myocytes.

### β-AR mRNA and Protein Expression in HF

**Figure 4:**
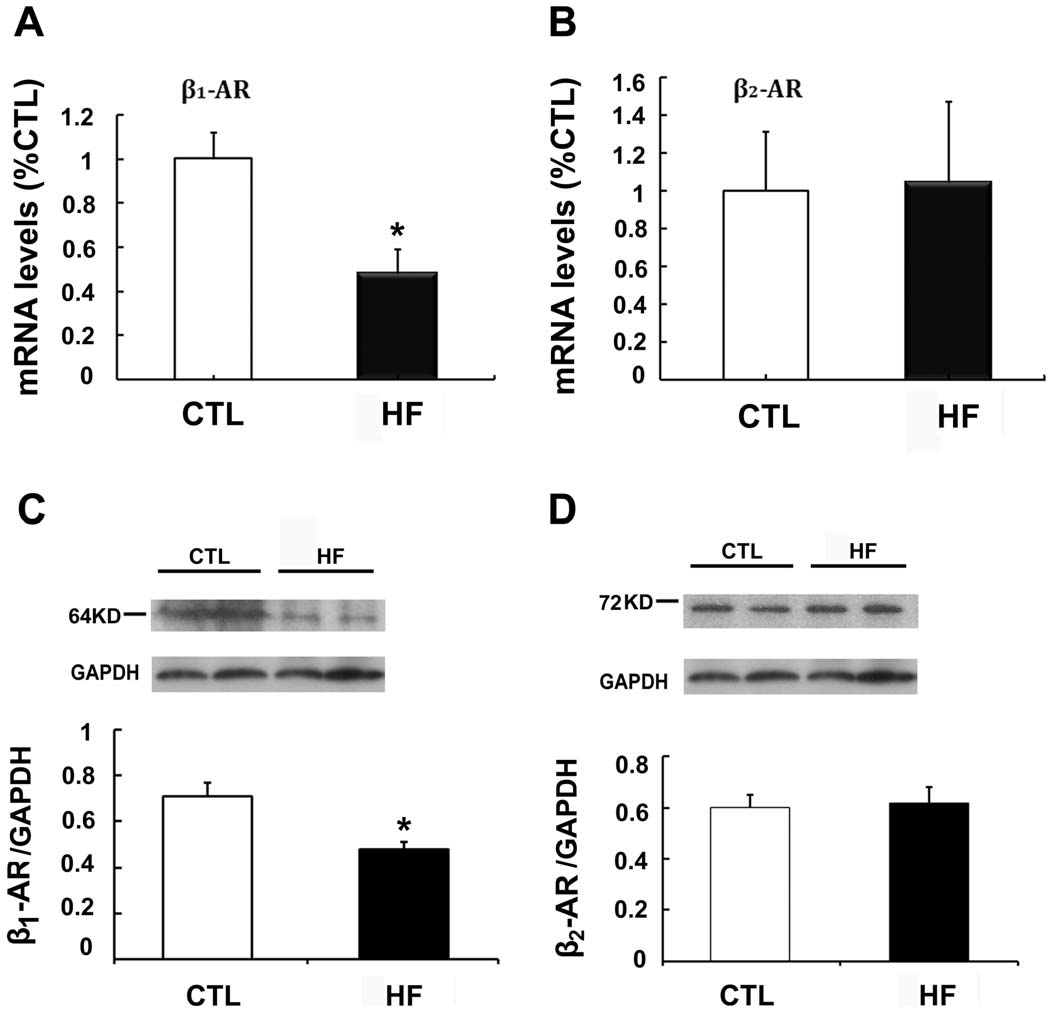
β_1_-and β_2_-AR mRNA and protein expression in heart failure (HF). (A) β_1_-AR mRNA level (n = 5, *p<0.05, HF vs.control, CTL). (B) β_2_-AR mRNA level (n = 5). (C) Representative protein bands of β_1_-AR in control and HF samples (upper); β_1_-AR protein expression (bottom, n = 5, *p<0.05, HF vs. CTL). (D) Representative protein bands of β_2_-AR in control and HF samples (upper); β_2_-AR protein expression (bottom).

Figure 4A and B show that β_1_-AR mRNA levels were reduced 52% in HF guinea pigs compared with controls (n = 5, p<0.05), whereas β_2_-AR mRNA levels remained unchanged. β_1_-AR protein showed a significant decline, as illustrated in Figure 4C. β_2_-AR protein remained unchanged (Figure 4D).

### I_Kr_ Inhibition Induced by β_2_-AR Stimulation Involves the cAMP/PKA Pathway

**Figure 5:**
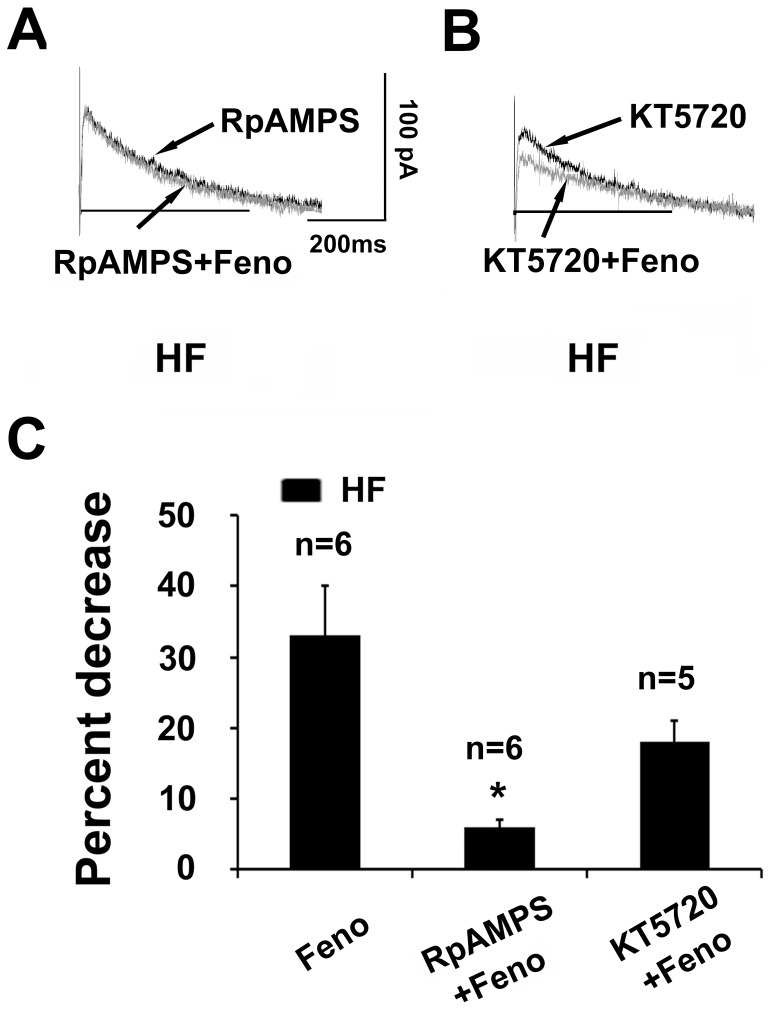
Effects of cAMP inhibition and PKA inhibition on I_kr_ response to β_2_-AR stimulation. (A) Superimposed tail current traces of I_kr_ recorded before and 10 min after application of 10 µM fenoterol (Feno) in the presence of RpCAMPS (100 µM) in pipette solution in a heart failure (HF) myocyte. (B) Superimposed tail current traces of I_kr_ recorded before and 10 min after application of 10 µM Feno in the presence of PKA inhibitor, KT5720 (2.5 µM) in a HF myocyte. (C) Summarized data for percent decrease in the amplitude of I_Kr_ tail current evoked by Feno alone, RpCAMPS plus fenoterol, and KT5720 plus Feno in HF myocytes (n = 6, 6, and 5 cells, 3 hearts, *p<0.05, RpCAMPS+ Feno vs. Feno). Current amplitudes were measured at the voltage of +40 mV.

It is known that the hERG K^+^ channel can be modulated by cAMP and PKA. β_2_-AR is coupled to the stimulatory G_s_ protein, which leads to the activation of adenylyl cyclase and production of cAMP. To examine whether cAMP activation mediates the I_Kr_ response to β_2_-AR stimulation, we investigated the effect of inhibitory cAMP analog Rp-cAMPS on the stimulatory action of fenoterol. As shown in Figure 5A and 5C, with intracellular application of Rp-cAMPS (100 µM), fenoterol–induced I_Kr_ decrease was almost fully prevented in HF myocytes (fenoterol alone, 33±7% decrease, n = 6; Rp-cAMPS, 6±1% decrease, n = 6; p<0.05). These results suggest that I_Kr_ inhibition induced by β_2_-AR stimulation involves cAMP activation.

We further examined the effect of PKA inhibition on the inhibitory effects of fenoterol. As illustrated in Figure 5B and 5C, fenoterol-induced I_kr_ decrease was largely abolished by pretreatment of HF myocytes with KT5720 (fenoterol alone, 33±7% decrease, n = 6; fenoterol plus KT5720, 18±4% decrease, n = 5; p = 0.051), supporting an involvement of PKA activation.

### Action Potential Duration (APD) is Prolonged by β_2_-AR Stimulation in HF Ventricular Myoctyes

**Figure 6:**
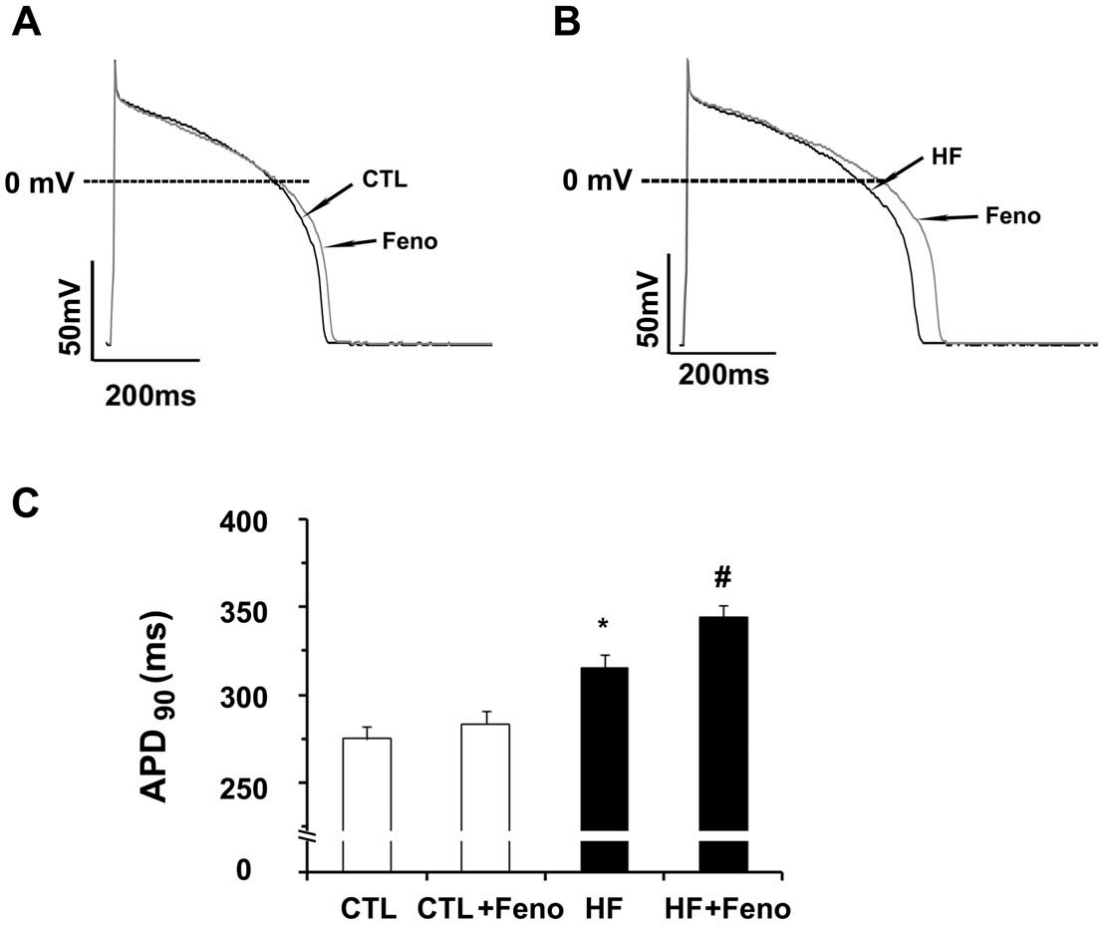
Effects of fenoterol (Feno) on action potential duration (APD). (A and B) Superimposed action potentials recorded before and 10 min after exposure to 10 µM Feno in a control and a heart failure (HF) myoctye of guinea pigs, respectively. (C) Summarized data for changes in APD at 90% of repolarization (APD_90_) by exposure to Feno in control (n = 8 cells, 3 hearts) and HF myocytes (n = 9 cells, 4 hearts, *p<0.01, HF vs. control; ^#^p<0.05, HF +fenoterol vs. HF).

Because action potential duration (APD) is dependent on the balance between depolarizing inward and repolarizing outward currents, an alteration in the amplitude of major repolarizing current such as I_Kr_ would lead to substantial changes in the repolarization process. We therefore examined the effects of fenoterol on AP in LV myocytes. Figure 6A and 6B shows the superimposed traces of AP recorded before and during exposure to fenoterol (10 µM) in control and HF myocytes. A summary of corresponding data is shown in Figure 6C. APD at 90% repolarization (APD_90_) was significantly prolonged in HF myocytes compared with control (315.1±8.1 ms, n = 8, vs. 274.8±7.3 ms, n = 9; p<0.01). Fenoterol caused significant prolongation of APD_90_ in HF myocytes (9.5%, 344.6±6.5 vs. 315.1±8.1ms, n = 8; p<0.05), whereas no significant prolongation of APD_90_ was observed in control myocytes (3%, 283.6±7.7 vs. 274.8±7.3 ms, n = 9). Fenoterol did not influence the AP amplitude and resting potentials in control and HF ventricular myocytes.

## Discussion

The present study demonstrates, for the first time, that increased response to β_2_-AR stimulation produces an inhibitory effect on I_Kr_ in ventricular myocytes of guinea pigs with descending aortic banding-induced heart failure, and this effect is induced by the activation of the cAMP/PKA pathway.

### Inhibitory Effect of β_2_-AR Activation on I_Kr_ in HF Ventricular Myocytes

The hERG channel (Kv11.1) underlies the rapid component of delayed rectifier potassium current (I_kr_) and is critical for the kinetics of cardiac AP repolarization [5], [14]. There is increasing evidence that hERG/I_Kr_ channels are modulated by various G protein-coupled receptors including α- and β-ARs, acting through the intracellular signaling modulators cAMP, PKA, and PKC [6], [7], [8], [9], [11]. It has been increasingly recognized that β_1_-and β_2_-AR coexist in the heart. In large mammal hearts, β_2_-AR may account for approximately 40% of β-AR in total. The reported ratio of β_1_-/β_2_-AR was 85/15 in guinea pig hearts [15]. Consistent with previous observations [16], in our HF guinea pigs, β_1_-AR was downregulated; however, β_2_-AR was unaltered. The increase of β_2_-AR density has been demonstrated in the LV of dogs with pacing-induced HF [17]. It has been demonstrated that in diseased ventricles, β_2_-AR stimulation exhibits a heightened response [12], [13]. Our results demonstrate, for the first time, that the responsiveness of I_Kr_ to β_2_-AR stimulation is enhanced in HF guinea pigs ventricular myocytes. The effect of selective β_2_-AR agonist fenoterol (10 µM) on I_Kr_ was far less effective in control myocytes; whereas, I_Kr_ was significantly inhibited by 10 µM fenoterol in HF myocytes. The reduction of I_Kr_ tail current occurred within 2–4 min and reached saturation about 10 min after addition of fenoterol into the bath. After washout of fenoterol, the inhibitory effect on I_Kr_ tail current was almost irreversible. In order to identify the β_2_-AR specific effect, we used salbutamol (10 µM), another highly selective β_2_-AR agonist, and achieved similar results (data not shown). Moreover, the inhibitory effect of fenoterol was almost completely abolished by β_2_-AR antagonist, but not by the β_1_-AR antagonist. Therefore, our findings indicate that the I_Kr_ inhibition induced by fenoterol is due to β_2_-AR activation.

Heart failure induced by pressure overload is a non-ischemic heart failure, which exhibits clinical and pathological features typical of hypertension-induced heart failure. Consistent with previous observations, myocytes from these hearts were enlarged. This model of heart failure is widely applied in the study of cardiac cellular electrophysiology. Sympathetic nervous system is activated in response to exercise and emotional stress, which results in increased levels of local and circulating catecholamines. Plasma norepinephrine levels of ∼ 3 µM and peak exercise norepinephrine levels of ∼ 15 µM have been detected in chronic HF in humans [18], giving our present findings utilizing 10 µM fenoterol a clinical relevance.

### cAMP/PKA Involvement in the Inhibitory Effect of β_2_-AR Activation on I_Kr_

Stimulation of different G protein-coupled receptors, including α_1_- and β-ARs, regulates I_Kr_ through intracellular messengers and provides a link between autonomic stimulation and cardiac repolarization [19]. Stimulation of β_2_-AR in the heart has classically been known to result in G_s_-mediated stimulation of adenylyl cyclase (AC), which leads to increased cellular cAMP, thus activating PKA [20]. It has been demonstrated in *the Xenopus laevis* oocyte expression system that PKA can reduce hERG currents via direct phosphorylation of all four putative PKA consensus sites [10]. In the present study, we demonstrated that fenoterol-induced inhibition of I_Kr_ was fully prevented by intracellular application of Rp-cAMPS, an inhibitory cAMP analog, suggesting that this effect may be mediated through a cAMP-dependent mechanism in HF ventricular myocytes. Furthermore, fenoterol-induced inhibition of I_Kr_ was partly attenuated by PKA inhibitor, implicating the involvement of PKA activation. The dual regulation of I_Kr_ by cAMP and PKA phosphorylation has been previously demonstrated [21]. hERG current may be decreased by cAMP-dependent activation of PKA; however, a putative direct binding of cAMP to the channel causes opposite effects. The net effect of dual hERG current regulation by PKA and cAMP is current reduction. A recent study has shown that β_2_-AR redistribution in HF can change cAMP compartmentation [22]. Our results do not exclude the possibility of direct binding of cAMP to the channel.

Cardiac β_2_-AR can couple with both G_s_ and G_i_ proteins. The complexity of the β_2_-AR signaling pathway, including functional compartmentalization of signaling mediated by G_i_, phosphatidylinositol 3-kinase, and mitogen-activated protein kinases (MAPKs), has been documented [20], [23]. Recently, Li *et al.* has reported that one or more A-kinase anchoring proteins (AKAPs) targets PKA to HERG channels and may contribute to the acute regulation of I_Kr_ by cAMP [24]. AKAPs are a structurally diverse group of proteins that lack primary structure sequence homology but share the function of localizing PKA to subcellular structures, substrates, and oftentimes with other members of the signaling pathway [25]. β-adrenergic signaling is maintained by the localization of PKA and phosphodiesterases to subcellular microdomains. In addition, in the rat brain, β_2_-ARs are found to be associated directly with Cav1.2 in a macromolecular signaling complex [26]. Indeed, the precise mechanism for β_2_-adrenergic stimulation-induced inhibition of I_Kr_ for HF myocytes remains to be further elucidated. For example, it remains unclear whether G_i_ pathway or a β_2_-AR-macromolecular signaling complex mediates the inhibition of I_Kr_ via β_2_-AR in HF ventricular myocytes.

### APD Prolongation by β_2_-AR Stimulation

One of the most characteristic electrophysiological remodeling in failing heart is APD prolongation, which is believed to mainly result from the downregulation of repolarizing outward potassium currents, including I_to_ (transient outward K^+^ current ), I_Ks_ and I_Kr_ in heart failure [27], [28]. In the present study, we found that QT interval was increased in failing guinea pigs and APD was prolonged in failing ventricular myocytes. Fenoterol caused significant prolongation of APD_90_ in failing ventricular myocytes, whereas no significant prolongation of APD_90_ was observed in control myocytes. The present study provides evidence that fenoterol-induced inhibition of I_Kr_ may result in delay in cardiac repolarization via stimulation of β_2_-AR in failing ventricular myocytes. Because the I_Kr_ is crucial for the repolarization of cardiac AP, inhibition of I_Kr_ induced by stimulation of β_2_-AR in failing ventricular myocytes may in part contribute to the delay in cardiac repolarization.Ventricular myocytes from the guinea pig heart possess a time- and voltage-independent Cl^−^ current induced by β-adrenoceptor activation [29], [30]. Activation of this current will result in outward current during the plateau phase of AP, shortening APD [31]. One limitation of the present study is that the AP experiments were performed without considering cAMP modulated Cl^−^ current. Therefore, the effect of fenoterol on cardiac repolarization should be studied without ignoring other currents in the future.

In conclusion, the inhibitory effect of I_Kr_ induced by β_2_-AR stimulation is increased in HF. The effect is likely to be mediated through a cAMP/PKA pathway mechanism in HF ventricular myocytes. This regulatory pathway of the β_2_-adrenergic system on I_kr_ current provides a possible relationship between stress and life-threatening arrhythmias in HF.

## Materials and Methods

All experiments were performed in accordance with animal care protocols approved by the Nanjing Medical University Institutional Animal Care and Use Committee.

### Heart Failure Model in Guinea Pigs

Heart failure was induced in adult male guinea pigs (250 to 300 g) by subtotal descending thoracic aortic banding. After sodium pentobarbital anesthesia (25 mg/kg, i.p.), the descending thoracic aorta was exposed sterilely through an incision in the left third intercostal space. A uniform degree of constriction around the descending thoracic aorta was produced by tightening a 2-0 surgical silk ligature around an 18-gauge needle. The needle was then withdrawn from the ligature, and the chest incision was closed. Sham animals underwent the same operation, with the exception that the aorta was not banded. Sham-operated animals were used as control. Aortic-banded animals and sham animals were housed and fed under identical conditions and were used 12 weeks after surgery. To monitor the progress of HF during the observation period, two-dimensional echocardiography (Sonos 5500, Hewlett Packard, Andover, MA, USA) was carried out periodically. ECGs and two-dimensional echocardiography were obtained under anesthesia (ketamine hydrochloride; 30 mg/kg, i.p.). The QTc was calculated by Bazett’s formula where QTc = QT/√RR. Following 12 weeks of aortic banding, guinea pigs showed signs of HF, including ventricular dilation, decreased ejection fraction (EF) and fractional shortening (FS), ascites, and pleural effusions.

### Myocyte Isolation and Electrophysiological Recordings

Single left ventricular (LV) myocytes were enzymatically dissociated from the heart of guinea-pigs (550–600 g) as described previously [6] with minor modification. Cardiomyocytes were transferred to a recording chamber that was continuously perfused with a bath solution. Pipettes had resistances of 3–6 MΩ after filling with the pipette solution. Following seal formation, access developed within 2–5 min and series resistance was compensated up to 80%. Whole-cell patch-clamp recordings were performed with an EPC-9 amplifier (HEKA, Germany). Flow rate through the chamber was maintained at 2–3 ml/min. I_kr_ was determined using the following test pulse protocol: after a holding potential of −40 mV, test pulses were applied at various voltages from −40 to +40 mV (step width 20 mV, step duration 200 ms) prior to returning to −40 mV for tail current recording [6], [7]. I_Kr_ current was recorded at 37±0.5°C. Substance effects were investigated on tail currents after the test pulse was applied at +40 mV. Measurements were repeated every two min.

**Table 2:** Gene-specific primer sequences used for real-time PCR analysis in this study.

Gene	Forward primersequence	Reverse primersequence	GenBank
*ADRB1*	TTCTATGTGCCCCTGTGCAT	GTGAACACGCCCATGATGAT	EU332753.1
*ADRB2*	CATCGTCAACATTGTGCACG	TGCCCCTGGTGACAAACAT	AJ459814.1
*GAPDH*	ACCACAGTCCATGCCATCAC	TCCACCACCCTGTTGCTGTA	

*ADRB1* and *ADRB2* refer to β_1_-AR and β_2_-AR.

For AP recording, extracellular solution (in mmol/L) contained NaCl 137, KCl 5.4, MgCl_2_ 1.0, CaCl_2_ 1.8, glucose 10, and HEPES 10; pH was adjusted to 7.4 with NaOH. Pipette solution (in mmol/L) contained potassium aspartate 120, KCl 20, MgCl_2_ 2.0, HEPES 10, EGTA 10, Na_2_ATP 10; pH was adjusted to 7.2 with KOH. APs were evoked using whole-cell current-clamp mode by suprathreshold current pulse of 5 ms duration at the frequency of 1 Hz. APD was measured at 90% repolarization (APD90). APs were generated with the same amplifier and recorded at 37±0.5°C.

### Solutions and Drugs

Preparation of the solution was performed as described previously by Wang *et al*. [6]. Calcium currents were blocked by the addition of 10 µM nifedipine in the bath solution, and 10 µM chromanol was used to block I_ks_. Na_2_-ATP, EGTA, creatine phosphate, *L*-glutamic acid, HEPES, taurine, bovine serum albumin (BSA), nifedipine, isoproterenol, xamoterol, fenoterol, CGP20712A, ICI118551, and Rp-cAMPS were purchased from Sigma (St. Louis, MO), collagenase II from Invitrogen (Carlsbad, CA), and KT5720 from Merck KGaA (Darmstadt, Germany). Dofetilide, a specific blocker of I_kr_ or hERG, was kindly provided by Pfizer Inc (NY, USA). All other reagents were obtained from Amresco (OH, USA).

For stock solutions, chromanol and KT5720 were dissolved in dimethyl sulfoxide (DMSO) at a final concentration of 1 mM and stored at −20°C until use. Final concentration of DMSO was less than 0.5% in the bath, which exerted no effect on the currents measured.

### Real Time RT-PCR Analysis

RNA was extracted from 100 mg of LV tissue using the Trizol reagent (Invitrogen) according to the manufacturer’s protocol. RNA concentration was determined spectrophotometrically. Reverse transcription of 2 µg RNA was performed in a 20-µl reaction mixture using an RT kit (Takara Biotech). The *GAPDH* housekeeping gene was used as a reference for loading control. The primers for genes examined are shown in Table 2. Optimized PCR cycle parameters included 2 min at 95°C, 10 sec at 95°C followed by 10 sec at 95°C, 10 sec at 57°C or 55°C, and 45 sec at 72°C for 40 cycles. Standard curves were determined for PCR efficiency. Quantitative real-time (RT) polymerase chain reaction PCR) was performed using the Applied Biosystems 7500 Real-time PCR System (Applied Biosystems, Foster City, CA, USA) with SYBR Green I (Invitrogen). Relative mRNA expression was determined using the 2^−△Ct^ method.

### Western Blot Analysis

Membrane fractions were prepared as previously described [32]. Protein samples were separated by 10% SDS-PAGE. The separated proteins were transferred by electrophoresis to PVDF membranes. Membranes were blocked for two h with Tris-Tween buffered saline (TTBS) solution containing 5% nonfat dried milk and incubated overnight with primary antibody against β_1_-AR and β_2_-AR (both from Santa Cruz Biotechnology, Santa Cruz, CA). After washing, blots were incubated for one h with appropriate peroxidase-conjugated goat anti-rabbit IgG secondary antibody (Santa Cruz Biotechnology). Staining was determined with chemiluminescence and quantified using image acquisition and analysis software. All data were expressed relative to GAPDH staining for the same samples on the same gels.

### Data Analysis

The data were acquired using Pulse +Pulsefit V 8.53 and were expressed as mean ± SEM. Western blot band intensities were expressed after background subtraction as optical density units (ODUs) normalized against GAPDH signal intensity for the same sample. Data comparisons were made using Student’s *t*-test (paired or unpaired). Differences were considered statistically significant if p<0.05.
